# Lagrangian modelling reveals sediment pathways at evolving coasts

**DOI:** 10.1038/s41598-025-92910-z

**Published:** 2025-03-14

**Authors:** Bart van Westen, Matthieu A. de Schipper, Stuart G. Pearson, Arjen P. Luijendijk

**Affiliations:** 1 Department of Hydraulic Engineering, Faculty of Civil Engineering and Geosciences, Delft University of Technology, 2628 CD Delft, The Netherlands; 2 Hydraulic Engineering, Resilient Ports and Coasts, Deltares, 2629 HV Delft, The Netherlands; 3 Water Research Laboratory, School of Civil and Environmental Engineering, UNSW Sydney, 110 King Street, Manly Vale, NSW 2093 Australia

**Keywords:** Civil engineering, Sedimentology, Physical oceanography

## Abstract

Coastal regions face increasing pressure from climate change, sea-level rise, and growing coastal populations. This “coastal squeeze” threatens both the systems’ sustainability and their ecosystem services. Coastal changes depend on the distribution of sediment throughout the system, which evolves continuously through complex transport processes. While we can quantify net morphological changes, this alone provides incomplete understanding of coastal evolution as similar morphological states can result from vastly different sediment movement patterns. Coastline perturbations-deviations from straight coastlines ranging from beach cusps to headlands, deltas, and artificial nourishments-exemplify this challenge. Although their diffusive morphological evolution is well understood, we have limited knowledge of the underlying sediment movement patterns driving this change. This study reveals how coastline perturbations alter sediment transport by tracing particles from origin to destination using Lagrangian tracking at the Sand Engine mega-nourishment. Our results demonstrate that perturbations alter both sediment dispersal and accumulation. During initial stages, the longshore dispersal of sediment is strongly restricted by rapid deposition and burial on both sides of the perturbation. A backward-tracing approach reveals that sediment deposition not only originates directly from the protruding part of the coastline, but also from updrift sources. As coastline perturbations diffuse over time, sediment movement patterns gradually converge toward those of an undisturbed coast. At locations with oblique wave incidence this evolution manifests itself with predominant downdrift dispersal and updrift trapping of sediment from adjacent beaches. The successful application of our Lagrangian approach to this multi-year evolution demonstrates the potential of sediment particle tracking for understanding more complex coastal environments. Increased understanding of sediment pathways enhances our ability to predict and communicate coastal response to interventions, supporting more effective management strategies.

## Introduction

Coastal regions face increasing pressure from climate change, sea-level rise, and growing coastal populations^1^. This “coastal squeeze” threatens both the systems’ sustainability and their ecosystem services^2^. Coastal systems provide numerous services to communities worldwide: recreational opportunities, unique habitats and biodiversity, protection against flooding, and drinking water provision^3–5^.

The provision of coastal services depends on the availability of sediment within the coastal system. The spatial distribution of sediment (e.g., sand) evolves over time through complex transport patterns, making sediment transport understanding crucial for effective coastal management^6–8^. Similar sediment redistribution along a coastline, or morphological change in general, can result from vastly different sediment pathways, a property known as equifinality^9–11^. Understanding net morphodynamic change alone, i.e., the description of morphological evolution through erosion and deposition, is therefore insufficient for comprehensively understanding coastal evolution - it requires the ability to trace sediment movement across the system^12,13^. This means not only quantifying how much sediment is redistributed but also tracking which sediment goes where, enabling better understanding and communication of causal relationships in coastal evolution.

The ability to map such sediment pathways has important practical applications, from finding sources of harbor sedimentation^14,15^ to tracking the dispersal of contaminated sediments from dam removals^16^ or dredged material from navigation channels and harbors^17^, and evaluating the effectiveness of nutrient-enhanced dune nourishment^18^. For example, coastal managers choosing an offshore disposal site for contaminated sediment need to evaluate how particles spread. While traditional Eulerian models can capture changes in bed levels or overall sediment budgets, they provide limited insight into whether sediments from a specific source migrate towards an area of interest. By building on widely used coastal models as a post-processing step, a Lagrangian approach uncovers these source-to-sink connections with minimal extra computational effort, offering a more direct way to inform management decisions.

Coastline perturbations, where the shoreline locally extends further seaward compared to its surroundings, provide a clear example where morphodynamic change alone cannot provide the complete explanation of coastal system evolution. These perturbations manifest across various spatial and temporal scales, occurring both naturally and anthropogenically, such as beaches on open coasts^19^, near tidal inlet systems^20,21^, river mouths^16^, coastal structures^22,23^, deltas^24–26^, and artificial nourishments^27–30^.

While the diffusive evolution of coastline perturbations is well-documented^31^, existing descriptions predominantly focus on the net morphological response. Numerous studies have quantitatively described the erosion of the coastal sections protruding from the surrounding and deposition on their flanks^31^, yet the underlying sediment pathways remain largely unexplored. Due to morphological equifinality, this morphodynamic behavior can result from a range of sediment pathways. Therefore, to develop a more comprehensive understanding of the coastal response to coastline perturbations, we need to know how sediment movement is affected by their presence, rather than merely describing the resulting morphology. How far does the sediment from a perturbation spread alongshore, or is it primarily restricted to the observable diffusive region? And is the observed sediment depositions in the vicinity of the perturbation all originating from the perturbation, or does it include contributions from more distance shores sources?

The ambiguity between sediment transport and morphological development is not limited to coastline perturbations. Similar challenges arise in understanding breaker bar or sand wave evolution^32^, and subaerial landform development^33^. Conventional Eulerian modeling approaches tabulate sediment fluxes on a grid of fixed points, and are insufficient to reveal the actual sediment pathways underlying the cumulative net response. By adopting a Lagrangian perspective, we can complement existing methods by simulating individual particle movements, thereby unraveling the sediment movement driving observed morphological changes.

Tracing sediment pathways requires complementing conventional Eulerian descriptions with Lagrangian analysis. However, mapping Lagrangian pathways poses significant challenges. Physical tracers^14,17,34–48^ can track sediment movement but have limitations: restricted spatiotemporal scales and labor-intensive procedures^38,40,49^. While numerical Lagrangian sediment methods exist^50–54^, their timescale of application is typically an order of magnitude smaller ($\sim$ months) than required for analyzing large-scale morphodynamic systems. They also generally neglect the trapping of sediment in regions with large deposition caused by morphodynamic evolution.

This study aims to understand the influence of coastline perturbations on longshore sediment movement by tracking particles across the coastal system. Through forward and backward tracing approaches, we address the following research question: How do coastline perturbations influence sediment pathways? Specifically, we focus on: (i) the dispersal of eroded sediment from perturbations along the coastline, and (ii) the source of deposited sediment on perturbation flanks, which is indicative of the mechanisms driving sediment accumulation.

We present a novel SedTRAILS-based approach^53^ for tracing sediment movement with a Lagrangian framework. We apply our Lagrangian approach to simulate sediment pathways at the Sand Engine mega-nourishment^28^ over multiple years of morphodynamic evolution. The Sand Engine presents an ideal case study for understanding coastal diffusion processes. Implemented in 2011 as a large artificial sediment pulse to nourish the Delfland coast over multiple decades^28^, it represents a “pure” coastline perturbation, with evolution less convoluted than natural examples. The Results section presents two complementary analyses: a forward-tracing study revealing how sediment from the man-made perturbation disperses over time (i), and a backward-tracing investigation identifying the origins of sediment that was observed to accumulate at nearby coastal sections (ii). In the Discussion, we examine the broader implications of our findings for understanding coastline perturbations on sediment movement and evaluate the potential of Lagrangian approaches in large-scale morphodynamic systems. After summarizing key insights in the Conclusions, we detail our Lagrangian framework in the Methods section.

## Results

**Fig. 1:**
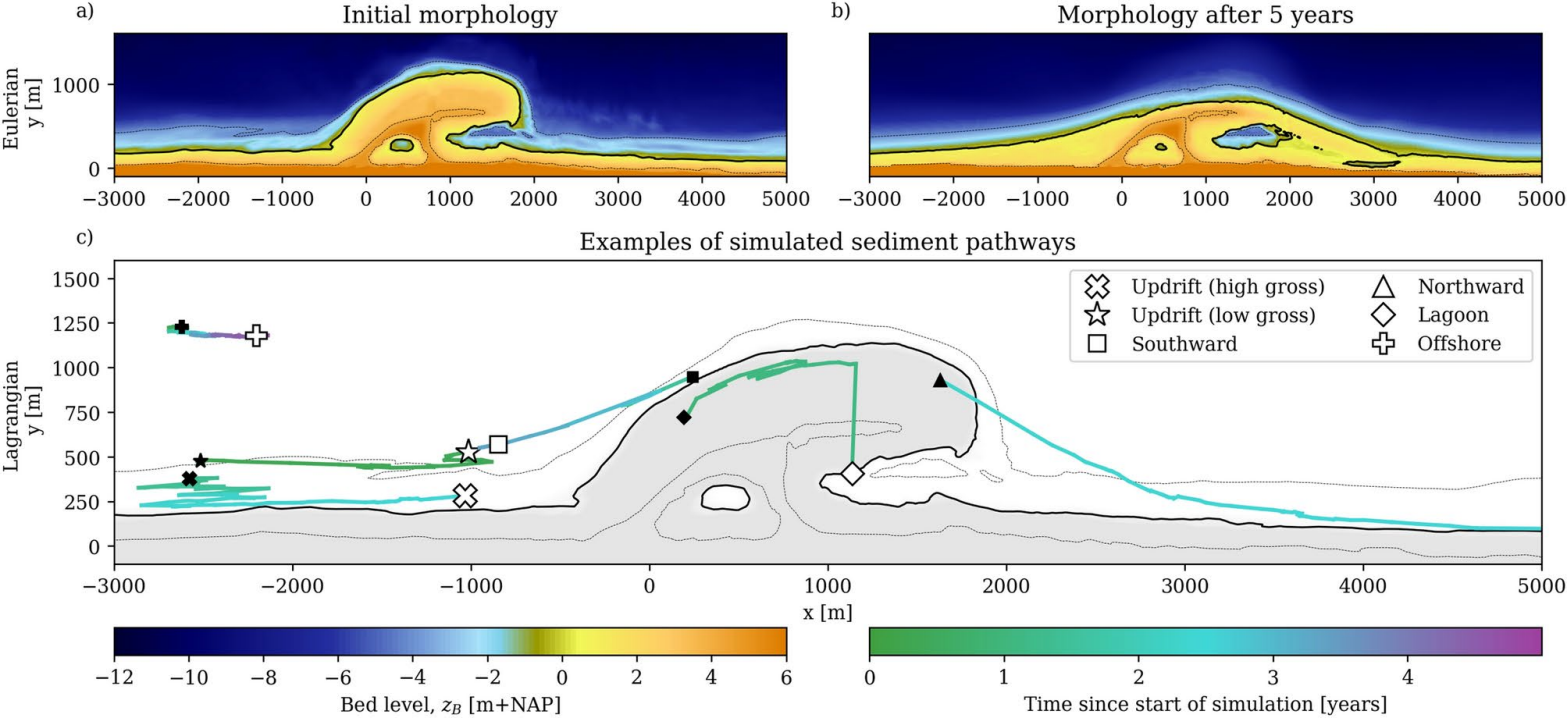
Sediment redistribution in the Eulerian (**a**,**b**) and Lagrangian simulation results (**c**). Panel (**a**) shows the initial Sand Engine morphology and panel (**b**) the morphology after 5 years of simulated development using a validated coastal area model^55^. Colors represent the bed level elevation relative to NAP (the Dutch reference for Mean Sea Level). Black lines indicate the 0 m+NAP (solid) and +/- 4 m+NAP (dashed) contours. Panel (**c**) shows the simulated trajectories of six selected sediment particles across the system, each represented by a different symbol. The origin of each pathway is shown as a filled marker, while the destination is shown as an open white marker. The color of the pathways indicates the time elapsed since the start of the simulation.

Our novel Lagrangian tool builds on a validated morphological coastal area model by van Westen et al. (2024)^55^, which integrates both marine and aeolian processes by coupling Delft3D Flexible Mesh^56^ and AeoLiS^33,57^ components. A 5-year brute-force hindcast, i.e., a simulation that captures the full period without schematizing or filtering conditions, is conducted, combining tides, wind, and waves^58^. The numerical model effectively reproduces areas of erosion at the most protruding parts of the coastline as well as sedimentation at adjacent beaches, resulting in a diffusive development of the Sand Engine nourishment (Fig. 1a,b). This Eulerian (grid-based, time-stepped) model is complemented by a Lagrangian post-processing method^53^, assuming a uniform sand grain size diameter. This combination can now reveal sediment pathways that were previously undetectable.

The resulting individual pathways (Fig. 1c) show significant variability in particle displacements, even when initial positions (e.g., particles with solid $\square$ and ◇ symbols) are close. These particles may be mobilized early in the simulation (greenish trajectories) or later (blue to purple trajectories) when the topography has evolved. Rapid displacements typically occur when particles are transported within the surf zone by wave-driven currents (e.g., $\triangle$) or by aeolian transport on the subaerial beach (e.g., ◇), while slower, gradual movement is observed further offshore (e.g., ). Although net particle displacements can be similar, with starting and ending points close to each other, substantial differences in gross movement patterns can be present(e.g., - ☆).

Of the 400,000 initial particles seeded in the coastal cell, nearly 40,000 were mobilized during the 5-year simulation. We distinguish between two types of particles in our simulation: native particles representing particles already present before construction and nourished particles placed during the Sand Engine’s construction. The large number of pathways enables volumetric analysis of the simulated Lagrangian particle movement. The combined sediment pathways, crossing the dry-wet interface, are analyzed to describe sediment movement along the coast.

### Alongshore dispersal of nourished sediment

**Fig. 2:**
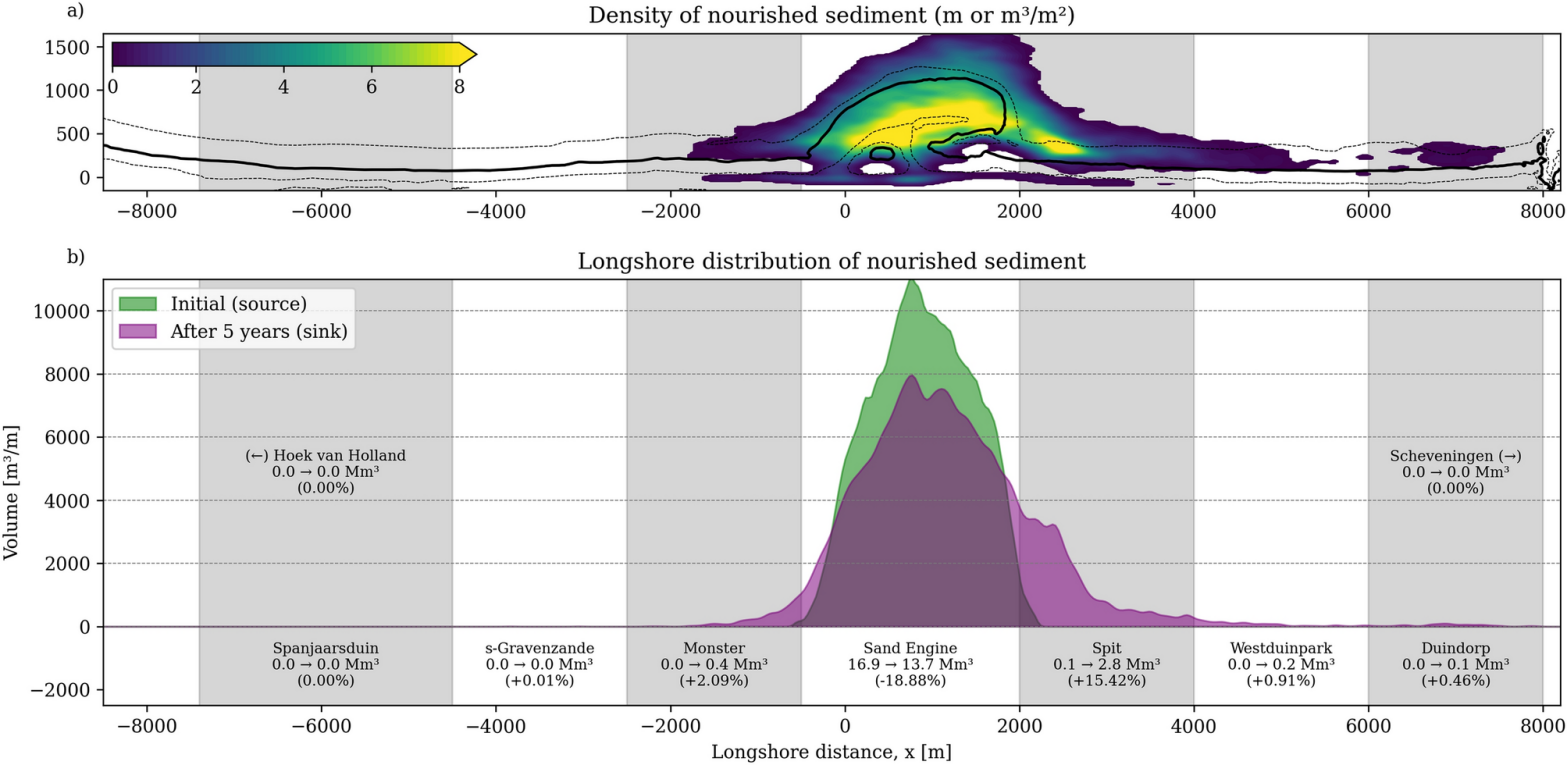
Longshore distribution of nourished sediment particles five years after construction. Panel a) shows a density map of the spatial particle distribution, with color indicating sand volume of the nourished sediment per surface area ($\hbox {m}^{3}$/$\hbox {m}^{2}$). Low density patches (blue) reach from the Monster beach section (x $\sim$ -2000 m) to Duindorp beach (x $\sim$ 7500 m), showing the longshore region over which the nourished sediment is spread. Panel b) shows the initial distribution of sediment particles associated with the nourishment (referred to as ‘source’) in green and the sediment distribution after 5 years in purple (‘sink’), highlighting the perturbations’ asymmetric diffusion with dominant northward transport. Longshore regions are labelled, and volumetric and percentage changes over time are provided.

Over the five years since construction, nourished sediment is computed to disperse in both directions along the shoreline (Fig. 2). Distinct asymmetry is visible in the sediment dispersal pattern and pathways, despite the more symmetrical transformation of the coastline, matching observations^59^. Driven by predominantly southwestern waves and asymmetrical tidal forcing, northward movement dominates: 3.0 $\hbox {Mm}^{3}$ of the nourished sediment moves north compared to 0.4 $\hbox {Mm}^{3}$ south, while 81% of nourished sediment remains within the Sand Engine placement area during these first five years. The spatial extent of dispersal also reflects this asymmetry. The computed southward dispersal reaches only approximately 2 km from the Sand Engine (Fig. 2, beach section called “Monster”). In contrast, northward transport extends further, and most transported sediment (15.4% of total nourished volume) is deposited between 2 km and 4 km at the northern side (“Spit” section). Smaller fractions reach 4 to 6 km (0.91% at “Westduinpark”) and 6 to 8 km (0.46% at “Duindorp”) from the nourishment. Based on the simulations, no sediment travels beyond Scheveningen harbor ($\sim$ 8 km north).

**Fig. 3:**
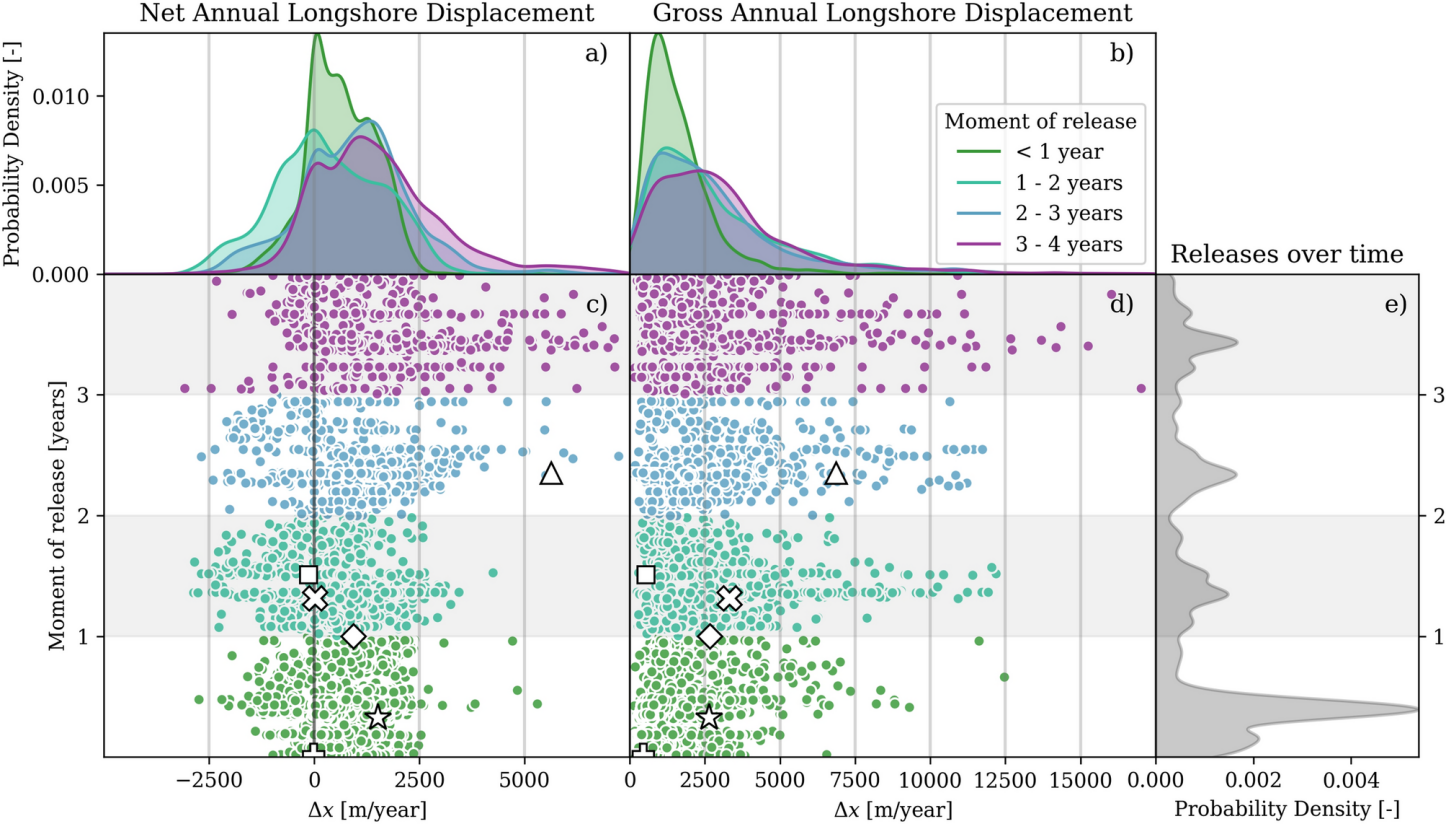
Evolution of longshore particle displacement in time and space. The scatter plots show one-year net (**c**) displacement distance (i.e., the Euclidean distance between release and burial locations) and gross (**d**) displacements (i.e., the distance travelled along the particle trajectory) of individual particles. The x-axis shows the displacement distance, and the y-axis the date of release, i.e., the moment of first mobilization. All particles are colored by release year. The northward shift in net movement shows the increase in asymmetric development over time, accompanied by generally larger net and gross longshore particle displacement. Selected particles from Fig. 1c are highlighted using the same symbols. Panel (**a**) and (**b**) present kernel density estimations (KDE) of displacement distributions for each year, using matching colors. Panel (**e**) shows temporal distribution of particle releases (number of initial mobilizations), with higher KDE values indicating more frequent mobilization events, particularly during the first storm season in the first year.

The characteristics of the longshore dispersal of sediment evolves as the Sand Engine’s morphology develops. To quantify this evolution, we analyze the one-year displacement of nourished particles from their initial mobilization, or release time. Figure 3c,d shows both net and gross longshore displacement, with particles grouped by their release year. For this particular analysis (i.e., Fig. 3), we exclude particles released in the final year, as they experienced less than a full year of movement before being truncated by the simulation’s end date. The figure also indicates the locations of representative particles from Fig. 1c using matching symbols (including both native and nourished particles).

Both net and gross longshore particle displacement increase as the Sand Engine evolves. The average net displacement more than doubles from 646 m for first-year particles to 1496 m for fourth-year particles, as shown by the green and purple markers in Fig. 3. Particles with the largest displacements also show this increase in displacement distance over time: the upper 5% of particles extend their northward reach from 1938 m in year 1 to 4335 m in year 4. Gross displacement distances follows a similar trend while on average being 2.9 times larger than the net displacement. Average gross movement increases from 1630 m in year 1 to 3124 m in year 4. The directional distribution of particle movement evolves significantly over time, transitioning from near-symmetrical movement in year 1 (i.e., skewness of Gaussian distribution $\gamma _1$ = 0.11), indicating dispersal to both sides, to strongly northward-skewed transport in year 4 ($\gamma _1$ = 0.89). As the Sand Engine evolves toward a straight, static coastline, both transport magnitude and directionality become stronger, indicating a stronger influence of the coastal perturbation during the initial phases of development.

### Morphodynamic-driven burial reduces longshore sediment dispersal

The shorter distances traveled by sediment during early stages result from rapid burial of nourished sediment at the Sand Engine’s accumulating flanks. This burial effect is particularly strong during the first year (narrow green KDE in Fig. 3a,b), when particles have less time to move before becoming trapped. As the Sand Engine evolves, the magnitude of bed level changes ($\Delta z_B$ [m]) decreases, enabling particles to remain mobile for longer periods. This evolution is reflected in burial statistics: the proportion of particles buried within their release year drops from 85% in year 1 to 55% in year 4.

Existing Lagrangian descriptions of sediment movement do not account for the influence of burial on particle dispersal. To quantify the importance of burial on pathway distance, we compare our modeled longshore dispersal with expected movement along an unperturbed coastline (equation (11) in Methods). In the surfzone, where wave-driven currents dominate, the transport rates ($Q_x$) are typically in the order of 300-700 $\hbox {m}^{3}$/m/year (Fig. 8a) and mixing layer thickness ($\delta _\text {mix}$) of 0.07-0.12 m (equation (5)), resulting in particle displacement estimates ($\Delta x_p$) from 2.5 to 10 km/year along an undisturbed coastline ranges. Simulated displacement around the perturbation shows much shorter distances, averaging only 200 m/year ($\sim$1000 m over five years), an order of magnitude below these theoretical estimates. As time progresses and morphodynamic activity decreases, particle movement gradually approaches these estimated transport rates. This convergence is evidenced by the increasing proportion of particles achieving significant northward movement: while less than 1% of particles traveled at least 2.5 km in year 1, this fraction grew to 21% by year 4.

**Fig. 4:**
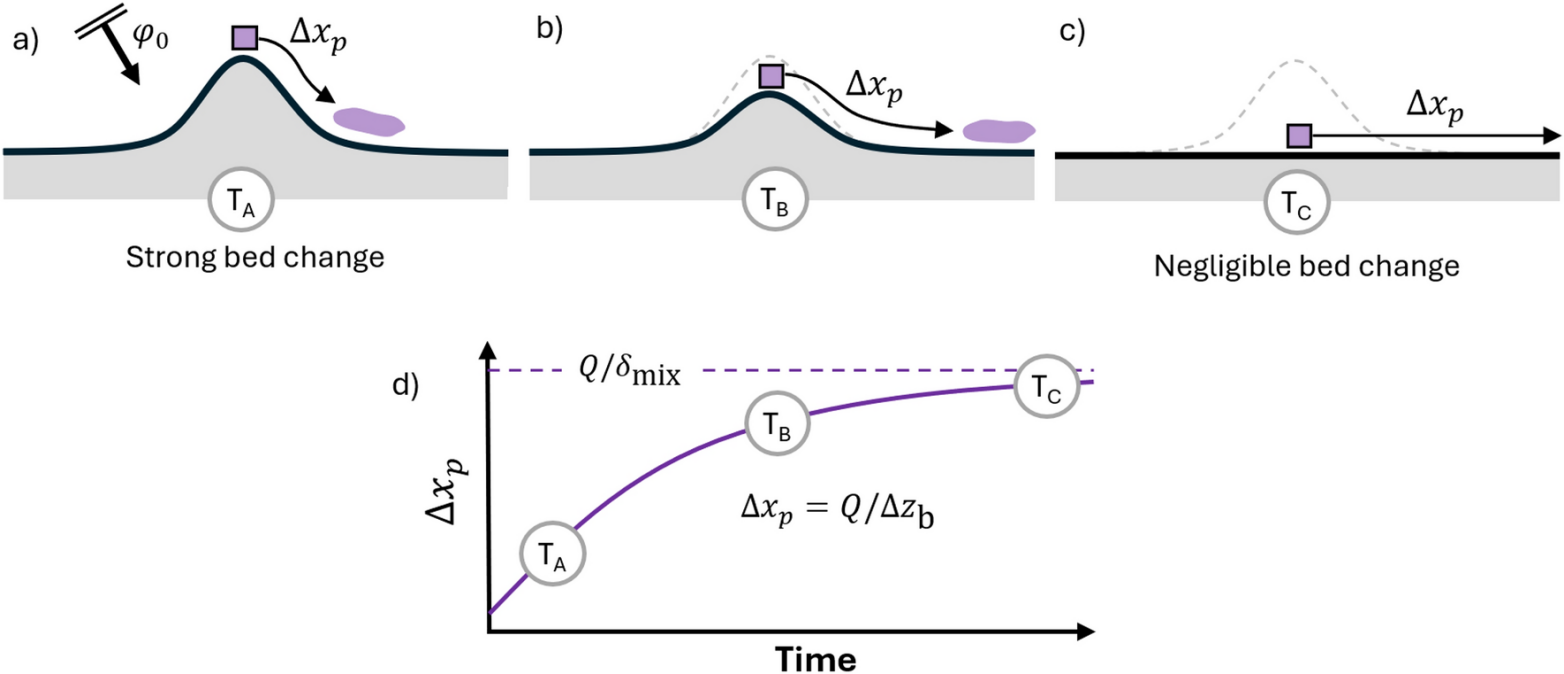
Morphodynamic-driven burial restricts longshore particle movement in accretive areas. Top panels show planform schematics of the transition from a burial-limited (panel **a**, **b**) to transport-limited (panel **c**) state. The corresponding temporal change of particle displacement is shown in panel (**d**).

Near perturbations, morphodynamic activity (O(1 m)) can significantly exceed mixing depth (O(10^-1^ m)), substantially constraining sediment movement (Fig. 4a,b). However, along straight, unperturbed coastlines where longshore transport gradients are minimal, morphodynamic activity and particle burial remain small. In these conditions, sediment dispersal is governed primarily by transport capacity and mixing layer thickness (equation (11), Fig. 4c). Conceptually, this can be captured in an adjusted displacement estimate for accretive coastal sections:

1 $$\begin{aligned} \overline{\Delta x_p}=\frac{Q_x}{\delta _\text {mix}} \min {(\Delta t , \Delta t_\text {burial})} = \frac{Q_x}{\max {(\delta _\text {mix}},\Delta z_\text {b})}\Delta t \end{aligned}$$

Where the $\Delta z_\text {b}$ indicates the magnitude of the accretion, and $\Delta t_\text {burial}$ the duration before a particle gets buried beyond the mixing layer depth. Using this adjusted formulation with typical values of $\Delta z_\text {b}$ ($\sim$ 5 m) we see indeed a nearly tenfold reduction in pathway distance. As perturbations diffuse over time and deposition rates decrease, the system gradually transitions from this burial-limited state toward transport-limited conditions characteristic of natural coastlines (Fig. 4d). Note that this only applies for accretive zones. In contrast, erosion of the bed could free particles that would otherwise have been trapped permanently, thus increasing the particle displacement.

### Direct and indirect effects drive sediment accumulation around perturbations

Tracing sediment origin at accumulating flanks reveals mechanisms underlying the diffusive evolution of coastline perturbations that are not apparent from morphological observations alone. While conventional understanding might suggest that flanking accretion results simply from sediment dispersing in both directions, our particle tracking analysis reveals a more complex reality. Sediment accumulation may occur from two distinct sources: the “direct” effect of sediment accumulation with sediment from the perturbation that is relocated and the “indirect” effect of sediment from the adjacent coastlines that is deposited due to the presence of the perturbation and the altered transport gradients.

**Fig. 5:**
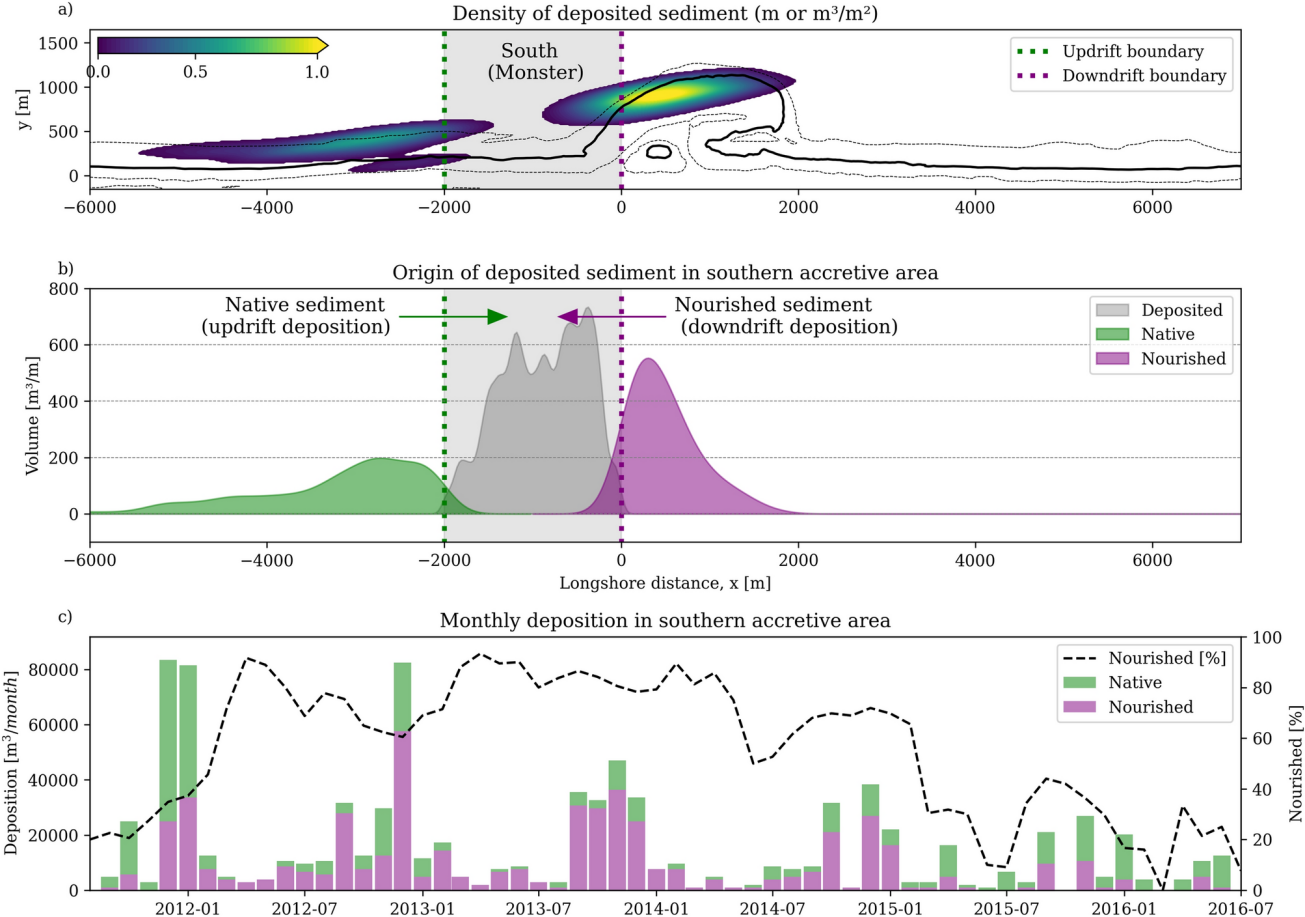
Sediment pathway analysis leading to deposition on the southern side of the Sand Engine coastline perturbation. Panel (**a**) shows the origin of sediment deposited in an area south of the Sand Engine (hatched control area called “South”), with colors indicating sediment quantity ($m^3/m^2$). Panel (**b**) shows the longshore distribution of this sediment. Bi-directional accumulation is indicated by sediment originating from updrift (green) and downdrift (purple) sources. Panel (**c**) quantifies the temporal contribution of these updrift and downdrift sources to sediment accumulation, with the dotted black line showing the percentage of downdrift deposition (nourished particles) relative to total accretion. Sediment accumulation is bi-directional, although the majority of sediment in the first three years originates from the nourishment. As the shoreline straightens over time, the majority of accumulation then comes from updrift, lowering the contribution of nourished particles (<40%).

**Fig. 6:**
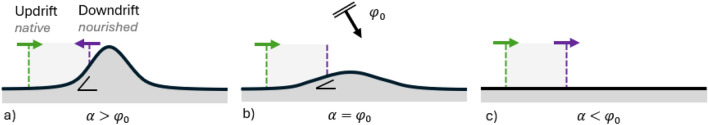
Evolving coastline gradients change the source of deposited sediment. Evolution of deposited sediment origin from bidirectional (**a**) to unidirectional accretion (**b**), to no net morphodynamic change (**c**), illustrating the relationship between coastline orientation $\alpha$, incident wave angle $\phi _0$, and resulting changes in transport patterns (illustrated with the colored arrows).

South of the Sand Engine, transport patterns show both of these contributions as the perturbation creates strong coastline gradients, enabling bidirectional transport. Figure 5a,b illustrates how the southern flank accumulates sediment through two mechanisms: directly from nourished sediment moving downdrift from the perturbation, and indirectly from native sediment arriving from updrift sources. This bidirectional transport emerges from the perturbation’s effect on coastline orientation in combination with the dominant sediment transport direction. The initial deviation of the shoreline orientation ($\alpha$) of up to $45^{\circ }$ with respect to the surrounding coast creates conditions for opposing transport patterns, as conceptually illustrated in Fig. 6a. In this region southward of the perturbation, on the updrift side relative to the dominant sediment transport direction on the perturbed coast, more than half of the accumulation does not originate from the perturbation itself. Especially during the first year, this results in a mixed accumulation pattern (Fig. 5c): 59% from updrift sources (native particles; green bars) and 41% from downdrift transport (nourished particles; purple bars).

Accumulation on the opposite northern side of the perturbation has a different source and ratio between nourished and native sediment. Being on the downdrift side of the dominant sediment transport direction, accretion follows a simple unidirectional pattern: nearly all deposited sediment (97%, Supplementary Fig. S1) originates from the perturbation itself, and creating a spit-like feature. The contrasting ratio between direct and indirect accumulation effects for the different sides demonstrates how accretion mechanisms may vary with position relative to both the perturbation and the predominant transport direction.

Over time, coastline perturbations can become less pronounced and the local coastline orientation with respect to the surrounding coast decreases. Conceptually, at some point, the system passes through a transition point where sediment transport becomes predominantly unidirectional $\alpha = \phi _0$ (Fig. 6b). This evolution manifests in the observed changing deposition patterns, as shown by the ratio of nourished particles contributing the accretion south of the Sand Engine (Fig. 5 c, black dotted line in). Downdrift deposition diminishes (from 70% in year two to 30% in year five) as the system returns to predominantly updrift deposition characteristic of an undisturbed coastline. As the coastline diffuses further, not all sediment is deposited at the flanks anymore. Due to reduced burial influence and smaller longshore gradients, particles start to bypass the perturbation. Ultimately, the coast becomes straight again and the amount of sediment entering the domain equals leaving it (Fig. 6c). This conceptual description is valid for coasts impinged by low-angle waves, and maybe differ in cases of high-angle waves^19^.

## Discussion

Understanding sand movement along the coast is vital for effective coastal management^6–8,12^. While Eulerian approaches^33,60^ are able to effectively capture net morphological change in coastal systems, they cannot reveal the sediment pathways driving this development. And, due to morphological equifinality, these sediment pathways can not be inferred from the coastline change alone. This makes net morphological change alone an incomplete descriptor of coastal evolution. Understanding coastal evolution therefore requires looking beyond these net changes, and the ability to map sediment pathways can provide this insight.

Most existing particle tracking approaches are constrained by morphostatic assumptions with no morphodynamic-driven burial^17,34,37–48^. These assumptions become particularly problematic when studying coastal perturbations that undergo significant morphological changes over multiple years, as demonstrated at our Sand Engine study site^59,61^. Physical tracer studies (e.g., with colored particles) would be ideal; however this technique faces similar challenges: the practical difficulty of tracking particles through evolving morphology challenges long-term pathway analysis^38,40^. Although geochemical approaches can indicate provenance of sediment at larger scales (O(10-100 km)^62^), these techniques may not have sufficient resolution to explain the evolution of coastal perturbations like the Sand Engine.

Our Lagrangian approach addresses these limitations through several key novelties. Using pre-computed Eulerian output reduces computational demands compared to calculating sediment velocities on a per-particle basis. By building upon frequently provided morphology and sediment transport fields from a validated Eulerian model^55^, we achieve multiple advances:

- The incorporation of morphodynamic-driven burial through simulated bed level changes extends Lagrangian modeling beyond traditional short-term analyses to multi-year studies of evolving coasts.
- The direct coupling between spatial patterns in mobilization probability and validated transport rates ensures Lagrangian movement aligns with Eulerian predictions. This produces realistic free-to-trapped ratios that naturally capture spatiotemporal variations in transport capacity and burial-induced particle trapping, eliminating the need for empirical parameters required in other approaches^51^.
- Our probabilistic approach to particle mobilization better represents the intermittent nature of sediment transport, particularly crucial for supply-limited aeolian processes^63^. While alternative approaches using reduced effective velocities^52,54^ offer computational advantages, they cannot capture the characteristic pattern of long burial periods punctuated by rapid, unidirectional movement.

The current method’s primary limitations stem from the accuracy of underlying Eulerian transport estimates. Any limitations in the Eulerian input are inherited by the simulated pathways. The Lagrangian method does not provide validated pathways (doing so would require a physical tracer study, which can be challenging and cost-prohibitive^38,40^), and thus should be considered as an alternative representation of Eulerian model results. This restricts the application range of the Lagrangian approach and the conclusions drawn within our study. In particular, cross-shore wave-driven and aeolian processes are only partly understood and underrepresented in the Eulerian results. Insufficient resolution of subgrid and intrawave processes in process-based morphodynamic models is known to result in unrealistic cross-shore morphodynamics, including cross-shore smoothing of surfzone bars^64^. To mitigate these effects in the Eulerian model, cross-shore transport was deliberately reduced^55^. Additionally, the Eulerian model underrepresents sediment exchange across the intertidal area^55^ likely due to missing swash-driven processes^65,66^. These limitations restricted us to draw conclusions primarily on longshore development, and prevent application in complex situations where morphological changes rely on cross-shore or intertidal processes. Since we have confidence in the bulk longshore transport rates, as the resulting morphological change compared well with observations, it is reasonable to assume that the simulated longshore particle movement is a representative depiction of real-world pathways.

On the contrary, these inherent limitations also provide valuable insights. By revealing specific shortcomings in Eulerian models through particle tracking, our approach identifies areas where conventional morphodynamic models need improvement. The utility of Lagrangian approaches ultimately depends on the quality of underlying Eulerian transport predictions. As morphodynamic modeling capabilities advance, the application range of particle tracking will expand accordingly.

This study employs a single-fraction in the Lagrangian analysis, aiming to capture the bulk of sediment transport. This single-fraction setup is consistent with the validated, observed morphological evolution over this five-year period. Finer fractions of natural sediment are likely to be mobilized faster and transported over longer distances than coarser fractions. Moreover, sediment hiding and exposure by coarser grains may influence the behaviour for forcing conditions close to the threshold of motion. A multi-fraction framework could provide further insight into sediment dynamics in these more complex settings. However, this requires modifying both Eulerian models to account for multiple sediment fractions, falling outside the scope of our study. Note that subaerial armouring processes are accounted for in the Eulerian fluxes from the AeoLiS model, by increasing the threshold for sand mobilization as the proportion of non-erodible grains rises and thus affect bulk sediment transport.

Our results have implications for coastal management, as the Lagrangian analysis reveals that even seemingly simple morphological evolution can mask complex sediment transport dynamics. While the Sand Engine exhibits relatively straightforward diffusive behavior at the coastline scale, individual particle trajectories show convoluted and diverse pathways (Fig. 1). Sediment starting from identical positions can reach vastly different destinations, and sediment with similar net displacements often follow different trajectories, as evidenced by varying gross-to-net displacement ratios. Sediment burial further complicates these patterns by intermittently restricting movement and influencing the timing of particle mobilization and deposition.

These detailed transport patterns, inaccessible through conventional Eulerian modeling, provide unique insights into sediment sources, pathways, and sinks. Although the observed accretion on both sides of the man made perturbation might initially suggest near-symmetric transport of nourished sand, our results clearly show that such conclusions cannot be drawn solely from bed level changes. A pathway analysis like the one presented here is highly valuable for coastal management and environmental impact assessments, as it can reveal the source material for harbour basin infilling^15^ or define the influence zones of (contaminated) sediment deposits^17^. This capability can also extend beyond traditional morphological response analysis by revealing the fate of specific sediment fractions, making it valuable for coastal management applications such as evaluating sediment release from dam removals^16^, and optimizing the placement of nutrient-enhanced sediment for dune development^18^. Backward tracking (sink-to-source) revealed sediment origins (Fig. 5) that may challenge basic intuition based solely on morphological evolution. This capability enhances our understanding of coastal interventions by revealing complex transport patterns, such as distinguishing between feeder- and leeside-effects in shoreface nourishments^27,67^ and unraveling sediment exchange pathways near ebb-tidal deltas^21^.

Regarding computational efficiency, the main cost is associated with the Eulerian model, while Lagrangian analysis adds relatively little overhead. Moreover, because each particle’s trajectory is independent, the additional Lagrangian analysis is straightforward to parallelize, making it scalable for larger coastal systems.

Overall, Lagrangian simulation provide a more intuitive visualization of complex sediment transport processes compared to aggregated vector fields, for both scientists and stakeholders alike. The approach opens possibilities for advanced analytical techniques previously unexplored at these spatiotemporal scales. Examples of sophisticated analysis techniques to reveal information hiding in Eulerian model results are the identification of Lagrangian Coherent Structures^68–71^, analysis of stratigraphic development^72^, quantification of sediment residence times^73,74^, and connectivity analysis^53^.

## Conclusion

This study advances our understanding of how coastal perturbations affect sediment pathways by revealing the mechanisms controlling both dispersal and accumulation patterns. Through novel Lagrangian analysis based on Eulerian model predictions applied to the Sand Engine mega-nourishment - chosen for its relatively simple diffusive evolution - we traced individual sediment pathways to uncover how perturbations impact sediment movement along the coast and thus the coastal evolution.

As coastline perturbations diffuse, their sediment disperses along the coast. Our analysis of the Sand Engine reveals that nourished sediment initially moves in both directions, while movement is being constrained during these early stages. Strong morphological change leads to rapid deposition on the perturbation flanks. Under these conditions, burial reduces particle displacement distance by an order of magnitude compared to undisturbed coastlines. As the perturbation size decreases, the system gradually transitions to transport conditions similar to the unperturbed coast, which is evidenced by increasing sediment displacement distances.

Coastline perturbations introduce strong gradients in coastline orientation, creating complex patterns of sediment accumulation driven by two distinct mechanisms. First, sediment can accumulate through direct supply, where material from the perturbation itself moves downdrift. Second, the perturbation’s presence modifies regional transport patterns, leading to indirect accumulation through updrift deposition. Model simulations revealed this dual mechanism during the Sand Engine’s initial phase, where accretion south of the perturbation showed near equal contributions from nourished sediment moving downdrift (direct supply) and native sediment arriving from updrift sources (indirect response). As the perturbation diffuses and coastline gradients decrease, the system transitions back to unidirectional transport characteristic of unperturbed coastlines.

These insights were enabled by our Lagrangian approach that builds upon validated Eulerian morphodynamic model results. By incorporating morphodynamic-driven burial and directly relating particle mobility to sediment transport rates, the method reveals sediment pathways impossible to detect through conventional Eulerian approaches. The method extends Lagrangian analysis beyond traditional (sub-)monthly timescales to multi-year periods, enabling the study of morphodynamic systems. Through volumetric comparison with Eulerian results, we verified that the approach accurately represents sediment transport and redistribution patterns provided by the coastal area model.

Our findings have broad implications for coastal management and research. While conventional approaches reveal net sediment volumes (i.e., how much sand is moved), our Lagrangian approach enables tracking of individual sediment pathways (i.e., which sand goes where). Since similar morphological changes can result from vastly different sediment movement patterns (equifinality), describing only the morphodynamic response provides an incomplete image of coastal evolution. The ability to map sediment pathways enhances our understanding of, and capacity to communicate, coastal responses to both natural and anthropogenic perturbations. Although this study focuses on longshore transport, the framework shows promise for analyzing more complex coastal features such as tidal inlets, ebb-tidal deltas, and shoreface nourishments. Applying this approach to such features could improve our understanding of sediment movement throughout the coastal system and enhance the effectiveness and communication of management strategies.

## Methods

We present a Lagrangian framework to analyze sediment pathways derived from Eulerian transport fields. While we refer to “particles” when describing sediment movement, our method differs from particle models that directly simulate particle advection based on hydrodynamic forcing. Instead, we employ a post-processing approach that conducts Lagrangian analysis on Eulerian model outputs while preserving the physical meaning of sediment parcel velocities. We adopt this particle terminology to facilitate clear description and visualization of transport patterns.

### Eulerian model

Our Lagrangian analysis builds on results from a validated coupled morphodynamic model^55^ that combines nearshore (Delft3D FM^60^) and aeolian (AeoLiS^33,75^) processes at the Sand Engine mega-nourishment^28^. This model successfully reproduces observed morphological evolution in both subaqueous and subaerial domains^55^. While some cross-shore processes, particularly at the dry-wet interface, are not fully captured, the model provides realistic sediment transport rates for our primary focus on longshore sediment redistribution.

The Eulerian framework comprises an unstructured grid of 17,412 cells for the Delft3D FM domain and a structured grid of 513,300 cells (1740 $\times$ 295) for the AeoLiS domain. The AeoLiS domain covers the subaerial zone within the larger Delft3D domain. Model outputs are stored hourly over the five-year simulation period, yielding 43,800 timesteps with updated forcing conditions (see van Westen et al. (2024)^55^ for detailed model description).

### Lagrangian transport model

The Eulerian model outputs are used to compute hourly spatial fields of (i) sediment velocity, and (ii) probability of transport. At a later stage, these Eulerian vector and scalar fields are used for the Lagrangian particle computation.

Sediment transport rates from both Delft3D FM and AeoLiS are provided as fluxes ($\hbox {m}^{3}$/m/s) rather than velocities. Since particle velocities differ from ambient current or wind velocities ($U_c$), we compute particle velocities based on bed shear stress relationships. Our approach distinguishes between three transport modes: nearshore bedload and suspended transport, and aeolian transport. A particle may experience any (combination) of these three modes during a model timestep, with its total particle displacement calculated as the sum the contribution for each mode.

For nearshore transport, we follow the approach by Soulsby et al. (2011)^51^, taking the bedload particle velocity from^76^:

2 $$\begin{aligned} U_{\text {bed}} = 10 \cdot u_{\star _\text {m}} \left( 1 - 0.7 \sqrt{\frac{\theta _{\text {cr}}}{\theta _{\text {max}}}} \right) \end{aligned}$$

where $u_{\star _\text {m}}$ [m/s] is the mean friction velocity over a wave cycle, and $\theta _{\text {max}}$ and $\theta _{\text {cr}}$ [-] represent the maximum and critical Shields parameters, respectively. The ratio between bedload and current velocity is defined as $R_b = U_\text {bed} / U_c$. For suspended load, the velocity is computed as:

3 $$\begin{aligned} U_{\text {sus}} = \frac{U_\text {bed} (1 - B)}{\left( \frac{8}{7} \right) - B} \cdot \frac{\left( \left( \frac{8}{7} R_b \right) ^{8 - 7B} - 1 \right) }{\left( \left( \frac{8}{7} R_b \right) ^{7 - 7B} - 1 \right) } \end{aligned}$$

where the Rouse parameter $B = w_s / (0.4 u_{\star \max })$ describes the vertical distribution of suspended sediment. All parameters above are obtained from the Delft3D FM component of the coupled Eulerian model. For aeolian transport, particle velocity $U_\text {aeolian}$ follows Sauermann et al. (2001)^77^:

4 $$\begin{aligned} \frac{(\vec {v_\text {eff}} - \vec {U_\text {aeolian}})|\vec {v_\text {eff}} - \vec {U_\text {aeolian}|}}{u_{f}^2} - \frac{\vec {U_\text {aeolian}}}{2 \alpha |\vec {U_\text {aeolian}}|} - \vec {\nabla z_{B}} = 0 \end{aligned}$$

where $v_\text {eff}$ [m/s] is the effective wind velocity driving the grains, accounting for saltation layer feedback and depending on shear velocity $u_{*}$ [m/s] and its threshold $u_{*,th}$ [m/s]. The grain settling velocity $u_f$ [m/s] and bed slope $\nabla z_{B}$ [-] represent fall and gravity effects, respectively. The parameter $\alpha$ = 0.42 [-] acts as an effective restitution coefficient for grain-bed interaction. These parameters are obtained from the AeoLiS model component.

Particles alternate between “free” and “trapped” states, as particles can be buried within the bed. To capture this behavior, we compute transport probability (*P*) for each transport mode (suspended load, bedload, and aeolian transport). When particles are free ($P=1$), transport occurs at computed velocity; when trapped ($P=0$), velocity reduces to zero. For a particle to potentially become mobilized, first of all, it should be located within the mixing layer. The mixing layer thickness $\delta _\text {mix}$ represents the vertical extent over which particles are actively mixed during transport. In physical tracer studies, this mixing depth typically corresponds to the level containing approximately 80% of recovered tracers^37,38,48,78^. Following Bertin et al. (2008)^79^, we relate mixing layer thickness to bed shear stress:

5 $$\begin{aligned} \delta _\text {mix} = 0.41 \sqrt{\tau _\text {max}-\tau _\text {cr}} \end{aligned}$$

where $\tau _\text {max}$ and $\tau _\text {cr}$ [N/$\hbox {m}^{2}$] are the maximum and critical bed shear stresses obtained from Delft3D FM. We consider aeolian mixing negligible. For particles within the mixing layer, transport probability is determined by the ratio between sediment in transport ($\delta _\text {bed}$, $\delta _\text {sus}$, or $\delta _\text {aeolian}$) and sediment in the mixing layer ($\delta _\text {mix}$).

6 $$\begin{aligned} P_\text {i} = {\left\{ \begin{array}{ll} \delta _\text {i}/\delta _\text {mix} & \text {if } \delta _\text {burial} \le \delta _\text {mix} \\ 0 & \text {if } \delta _\text {burial} > \delta _\text {mix} \end{array}\right. } \end{aligned}$$

where subscript ’i’ represents either bedload, suspended load, or aeolian transport mode. These quantities are expressed as volumes per unit area [$\hbox {m}^{3}$/$\hbox {m}^{2}$] or equivalent thicknesses [m]. We compute the amount of sediment in transport by dividing the Eulerian transport fluxes [$\hbox {m}^{3}$/m/s] by their respective particle velocities [m/s]:

7 $$\begin{aligned} \delta _\text {i} = \frac{Q_\text {i}}{U_\text {i}} \end{aligned}$$

An important consequence of this approach is that particle velocity (*U*) has limited impact on collective movement patterns: higher velocities lead to more rapid movement of individual particles but at a lower transport probability, as these effects cancel each other. The total particle movement is ultimately governed by the transport rate (*Q*).

This probability-based approach differs from alternative methods that apply the ratio as a velocity reduction factor-where instead of moving at full speed 1% of the time, particles move continuously at 1% speed^54^. While this alternative works adequately in the nearshore domain, it proves problematic for aeolian transport. With typical aeolian pickup rates being significantly lower compared to nearshore pickup, particles are infrequently, but more directly, moved from the intertidal zone to the dunes when mobilized. A velocity reduction approach would instead produce unrealistically slow, continuous movement.

Having computed all Eulerian fields of particle velocities and transport probabilities at hourly intervals over five years, we simulate particle movement using a timestep ($\Delta t$) of 60 seconds. The total displacement of each particle is determined by:

8 $$\begin{aligned} X_p(t + \Delta t) = X_p(t) + \sum _{\text {i}} \int _{t}^{t + \Delta t} P_\text {i} \cdot U_\text {i}(x, y, t)dt \end{aligned}$$

where ’i’ represents the summation of the bedload, suspended load, and aeolian transport modes. The integration is performed using a fourth-order Runge-Kutta advection scheme. The particle burial changes over time with bed level changes:

9 $$\begin{aligned} \delta _\text {burial}(t + \Delta t) = \delta _\text {burial}(t) + \int _{t}^{t + \Delta t} \Delta z_{B}(x,y,t)dt \end{aligned}$$

where $\Delta z_{B}(x,y,t)$ represents the bed level change at the particle’s location $(x_p(t), y_p(t))$ at time *t*.

### Initial particle distribution

We initialize particles with uniform spatial distribution across both horizontal dimensions and depth. Particles are placed on an equidistant horizontal grid covering the entire Delfland coast (16.4 km $\times$ 1.8 km), with resolutions of 8.2 m and 9.0 m in the longshore and cross-shore directions, respectively. This grid comprises 2000 particles in the longshore direction and 200 in the cross-shore direction. Each particle is assigned a random depth of between 0 and 13 meters below the bed surface-corresponding to the maximum construction depth of the Sand Engine-ensuring coverage of the complete nourished volume. The particle grainsize is 250 $\mu m$, consistent with the settings of the Delft3D FM simulation^55^.

### Comparing Eulerian and Lagrangian transport

Eulerian and Lagrangian transport frameworks use different metrics: volumetric transport rates ($\hbox {m}^{3}$/m/s) and particle velocities (m/s), respectively. These approaches can be related through a well-established relationship^35,36,38,44,48,54,80,81^ between Eulerian longshore transport ($Q_x$ [$\hbox {m}^{3}$/m/s]) and Lagrangian tracer movement ($\overline{V_{x,p}}$ [m/s]):

10 $$\begin{aligned} Q_{x} = \overline{V_{x,p}}\times \delta _\text {mix} \end{aligned}$$

Here, $\delta _\text {mix}$ [m] represents the mixing layer thickness-the active surface layer where sediment particles are mobilized during transport (equation (5)). The longshore particle velocity $\overline{V_{x,p}}$ [m/s] describes the average particle movement, calculated from collective displacement over time ($\overline{V_{x,p}} =\overline{\Delta x_p}/\Delta t$). Rearranging these terms yields the average longshore displacement of tracers, $\overline{\Delta x_p}$ [m]:

11 $$\begin{aligned} \overline{\Delta x_p}=\frac{Q_x}{\delta _\text {mix}} \Delta t \end{aligned}$$

While this (cross-shore integrated longshore transport) formulation provides useful first-order estimates for simplified cases, our approach extends beyond these constraints by assigning volumetric dimensions to particles. By distributing 400,000 particles across 383,760,000 $\hbox {m}^{3}$, each particle represents 959.4 $\hbox {m}^{3}$ of sediment, enabling direct comparisons between Lagrangian movement and Eulerian redistribution patterns.

### Verification of simulated pathways

To validate our Lagrangian approach, we compare the simulated particle pathways with Eulerian transport patterns. While direct comparison with physical tracer measurements is not possible at our study site, the underlying Eulerian model shows good agreement with observed morphological evolution^55^. We focus our verification on two key metrics: volumetric sediment redistribution patterns and annual longshore transport rates.

**Fig. 7:**
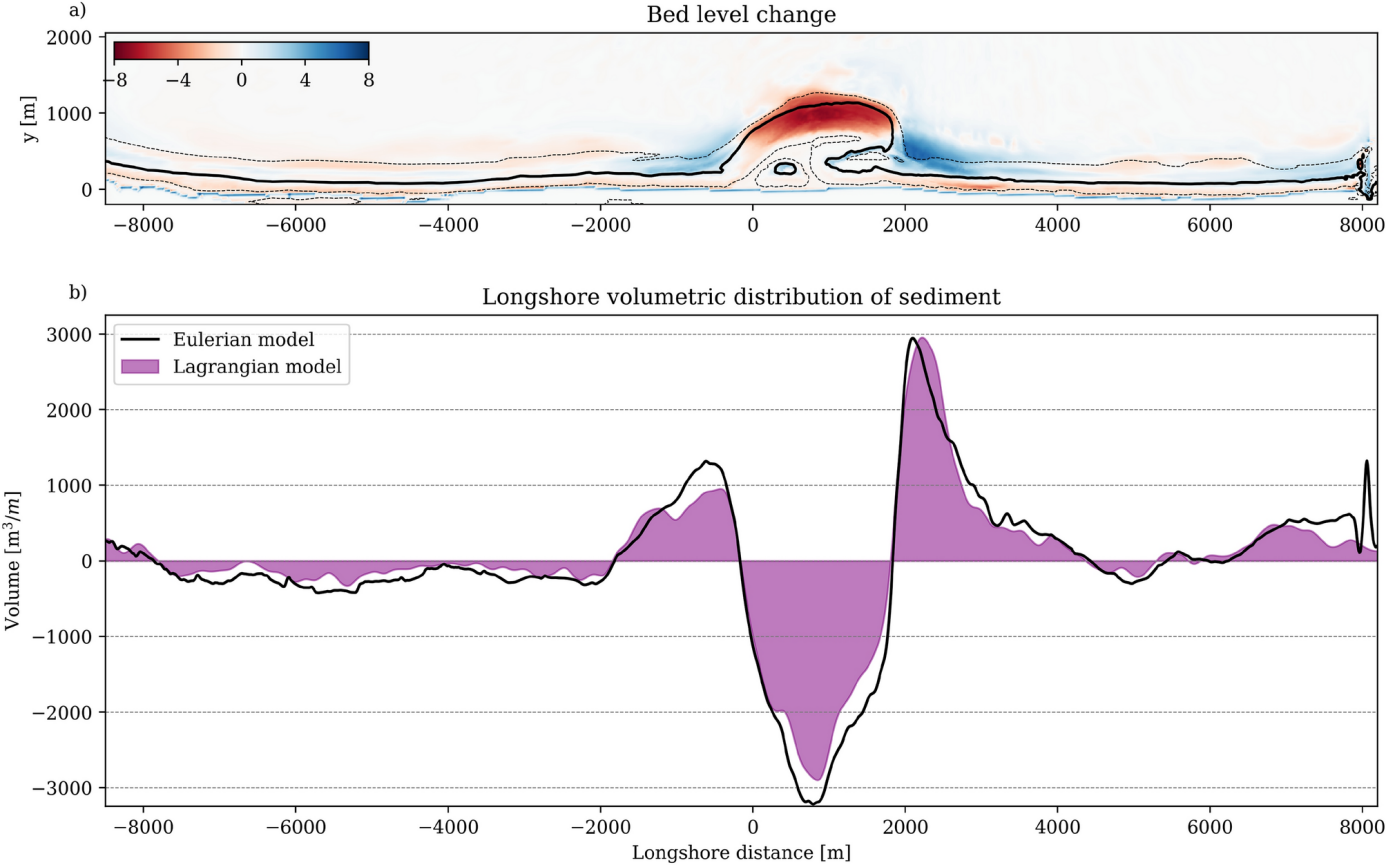
Verification of Lagrangian approach through sediment redistribution patterns. (**a**) Cumulative bed level change in meters after five years showing erosion (red) and accretion (blue) zones from Eulerian model. (**b**) Comparison between Eulerian-computed redistribution (black line) and Lagrangian-derived volumetric changes (purple patch) along the coastline, demonstrating agreement between approaches.

The five-year morphological evolution of the Sand Engine provides our first verification case. Eulerian results show erosion at the most seaward parts of the perturbation (red area in Fig. 7a) and accretion both north and south (blue areas). Quantitative analysis reveals maximum erosion of 3219 $\hbox {m}^{3}$ per m alongshore at the tip, accretion of 2943 $\hbox {m}^{3}$/m at the northern flank and 1000 $\hbox {m}^{3}$/m along the southern flank (black line, Fig. 7b). These patterns align well with field observations^55,59^.

Our Lagrangian approach, in combination with the particle seeding over a three-dimensional domain, enables volumetric comparison through particle tracking. Lagrangian particle movement from position A to B translates directly to volumetric Eulerian erosion at A and deposition at B. This particle-based redistribution (purple patch in Fig. 7b) shows good agreement with Eulerian predictions, although slightly underestimating erosion (2902 $\hbox {m}^{3}$/m) at the Sand Engine tip and accretion to its south (953 $\hbox {m}^{3}$/m).

**Fig. 8:**
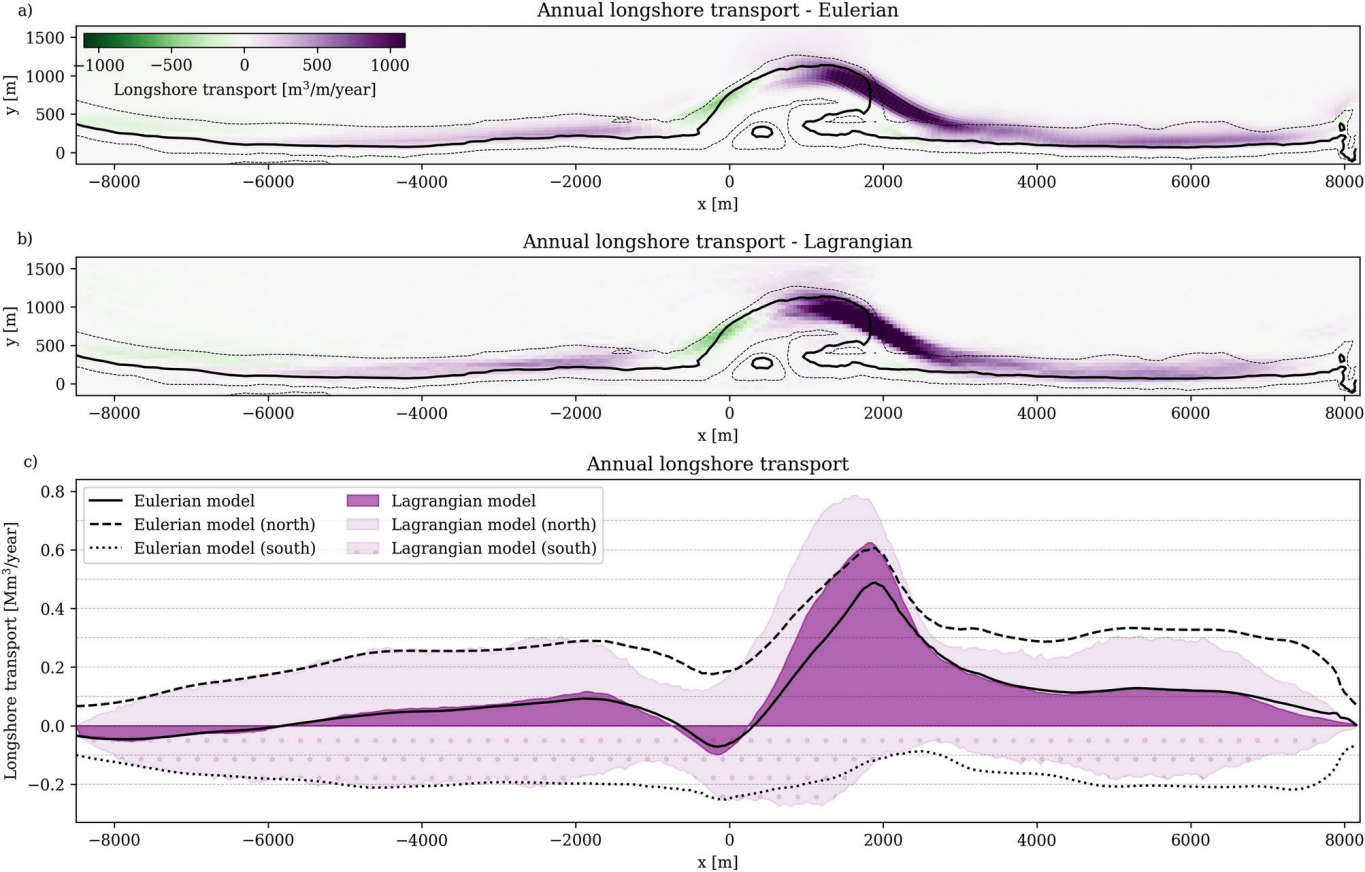
Comparison of the Eulerian (**a**) and Lagrangian (**b**) annual longshore transport rates. The longshore transport integrated over the cross-shore is shown in panel **c** for the Eulerian (black lines) and Lagrangian (purple patches) simulations.

We extend this verification to annual longshore transport rates, $Q_x$ [$\hbox {m}^{3}$/m/year]. Figure 8a presents longshore Eulerian transport in northward (purple) and southward (green) directions. For Lagrangian comparison (Fig. 8b), we divide the domain into a structured grid. We count the number of particles crossing the cell edges and multiply this number of particle crossings by the particle representative volume (959.4 $\hbox {m}^{3}$).

This comparison (purple patches vs. black lines in Fig. 8c) demonstrates good agreement in transport patterns, with a notable exception at the Sand Engine tip where Lagrangian transport rates (0.63 $\hbox {Mm}^{3}$/year) exceed Eulerian estimates (0.49 $\hbox {Mm}^{3}$/year). The discrepancy emerges at a location that is prone to the confluence of complex flow patterns, steep transport gradients, and rapid bed level changes, making it difficult to isolate the precise cause of the mismatch. Nevertheless, the final particle distribution (Fig. 7) still closely matches the Eulerian volumetric redistribution patterns. As our analysis focuses primarily on understanding sediment pathways and final distribution rather than longshore transport rates, these localized differences are considered to not significantly affect our main conclusions.

## Supplementary Information

Supplementary Information 1.

Supplementary Information 2.

## Data Availability

The software developed and applied during the current study is available from the corresponding author on request.
