# Supplementary material for: Lagrangian modelling reveals sediment pathways at evolving coasts
Source: Sci Rep. 2025 Mar 14;15:8793. doi: 10.1038/s41598-025-92910-z (PMC11906850; doi:10.1038/s41598-025-92910-z)
Supplement: Supplementary file 1 — Supplementary Information 1. [file 41598_2025_92910_MOESM1_ESM.docx]

Supplementary Figure 1: Sediment pathway analysis leading to deposition on the northern side of the Sand Engine coastline perturbation. Panel a) shows the origin of sediment deposited in an area north of the Sand Engine (hatched control area called "North"), with colors sediment quantity (m^3^/m^2^). Panel b) shows the longshore distribution of this sediment. Panel c) quantifies the temporal evolution of updrift (purple) and downdrift (green) sources to sediment accumulation, with the dotted purple line showing the percentage of downdrift deposition relative to total accretion.
