# Supplementary figures and images for: Lagrangian modelling reveals sediment pathways at evolving coasts
Source: Sci Rep. 2025 Mar 14;15:8793. doi: 10.1038/s41598-025-92910-z (PMC11906850; doi:10.1038/s41598-025-92910-z)

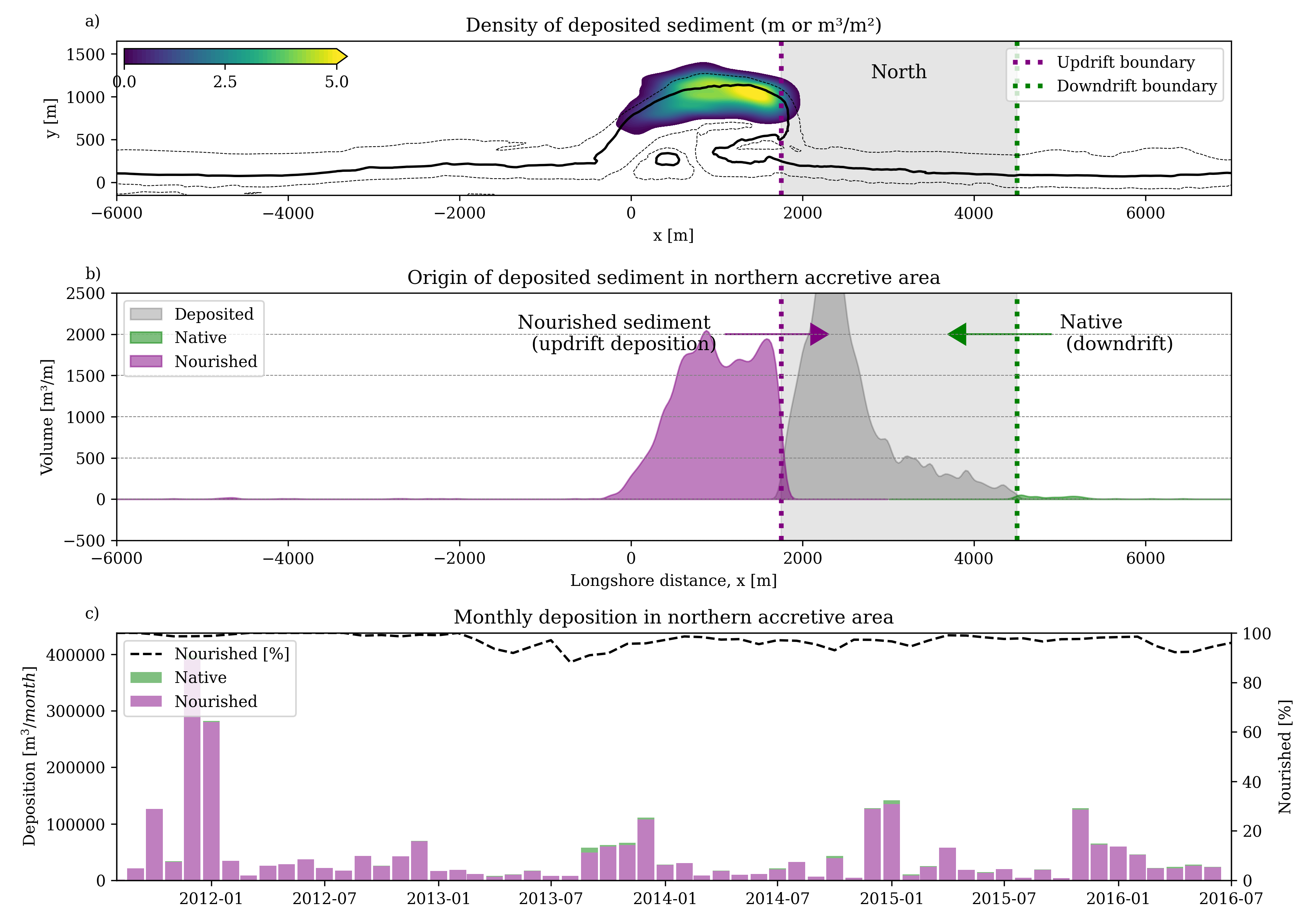

Supplement: Supplementary file 2 — Supplementary Information 2. [file 41598_2025_92910_MOESM2_ESM.png]
